# Targeted Medical Interventions for the Prevention and Treatment of Viral Hemorrhagic Fever: A Rapid Systematic Review of Updated Intelligence from the 74th Annual Meeting of the American Society of Tropical Medicine and Hygiene

**DOI:** 10.3390/tropicalmed11100296

**Published:** 2026-10-19

**Authors:** Gregory D. Hawley, Michael Klowak, Amanda K. Hepmel, Jamal W. Tarrabain, Chu S. Wang, Keshini Abeyewardene, Jahmar Hewitt, Asal Adawi, Candice Madakadze, Syed Z. Ahmad, Aquilla Reid-John, Andrea K. Boggild

**Affiliations:** 1 Institute of Medical Science, University of Toronto, Toronto, ON M5S 3H2, Canada; 2 Tropical Disease Unit, Toronto General Hospital, Toronto, ON M5G 2C4, Canada; 3 Department of Medicine, University of Toronto, Toronto, ON M5S 3H2, Canada; 4 Department of Family and Community Medicine, University of Toronto, Toronto, ON M5G 1V7, Canada; 5 Division of Infectious Diseases, Department of Medicine, University Health Network and Mount Sinai Hospital, Toronto, ON M5G 2C4, Canada; 6 College of Medicine and Health Sciences, Palestine Polytechnic University, Wadi Al-Haria, Hebron P.O. Box 198, Palestine; 7 School of Medicine, University College Cork, T12 AK54 Cork, Ireland; 8 Office of Access and Outreach, Temerty Faculty of Medicine, University of Toronto, Toronto, ON M5S 1A8, Canada

**Keywords:** ebolavirus, experimental therapeutics, filoviruses, hendra virus, high-consequence pathogen, marburg, nipah virus, sudan ebolavirus, viral hemorrhagic fever

## Abstract

**Background**: Viral hemorrhagic fevers (VHFs) are severe, often high-consequence, life-threatening illnesses characterized by systemic disease, hemorrhage, and high mortality. The American Society of Tropical Medicine and Hygiene (ASTMH) Annual Meeting functions as a major international forum for the presentation of emerging data on infectious diseases, including novel pharmacologic and biologic interventions for VHF. However, while ASTMH presentations provide timely and high-impact insights, there is no efficient, consolidated synthesis of this rapidly evolving evidence base. Given the acute, outbreak-prone nature of VHF and the need for rapid translation of emerging data into clinical and public health practice, a structured synthesis of these data is warranted. **Methods**: This systematic review follows PRISMA guidelines and is limited to presentations delivered at the ASTMH Annual Meeting in Toronto (9–13 November 2025) in order to capture evolving evidence as it has emerged. Studies of all designs evaluating vaccines, chemoprophylactic agents, or targeted biological therapies for the prevention or treatment of VHF in adults and children were included. Data were extracted on incidence of VHF among exposed populations, safety, toxicity, and tolerability of preventive and therapeutic interventions, and morbidity and mortality outcomes among treated individuals. Secondary outcomes include hospitalization length-of-stay, economic outcomes, and measures of feasibility, acceptability, accessibility, and health equity. **Results**: Nine studies (four oral presentations, two poster presentations, and three hand-searched references) were included in the final synthesis. Seven studies evaluated pharmacologic preventive interventions and two evaluated experimental pharmacotherapeutics, with two studies contributing evidence to both categories. One observational study and one narrative review provided contextual evidence on vaccine readiness and outbreak preparedness. **Discussion/Conclusions**: This systematic review provides a rapid, structured synthesis of targeted medical interventions as emerging evidence at an expert scientific venue, while addressing the current lack of a consolidated, accessible summary of emerging VHF evidence. By rapidly organizing and evaluating these data, this work facilitates timely interpretation and application in clinical and public health settings where rapid decision-making is critical.

## 1. Introduction

Viral hemorrhagic fevers (VHFs) are a group of high-consequence, life-threatening febrile illnesses caused by RNA viruses belonging to various viral families [1]. They encompass pathogens with different geographic distributions, reservoir hosts, modes of transmission, and epidemiology, including filoviruses (e.g., Ebola and Marburg viruses), arenaviruses (e.g., Lassa virus and New World arenaviruses), bunyaviruses (e.g., Crimean–Congo hemorrhagic fever virus and Rift Valley fever viruses), and flaviviruses (e.g., yellow fever and dengue) [2]. While VHFs vary with regard to severity and clinical presentation, they produce overlapping clinical manifestations, namely a shared systemic involvement, vascular dysfunction, and high mortality relative to non-hemorrhagic fever viruses [3]. The clinical course of VHF generally begins with non-specific symptoms such as fever, headache, myalgia, abdominal pain, and vomiting. As it progresses, patients may develop hemorrhagic manifestations of disease including petechiae, ecchymoses, hematemesis, coagulopathy, and thrombocytopenia [1,4]. Once severe manifestations develop, the disease can rapidly progress to jaundice, shock, altered mental status, and multi-organ failure, associated with significant morbidity and mortality. Management can thus be particularly challenging in outbreak settings where diagnostic and therapeutic resources are limited [2,5].

The Annual Meeting of the American Society of Tropical Medicine and Hygiene (ASTMH) functions as a major international forum for the presentation of emerging data on infectious diseases, including novel pharmacologic and biologic interventions for VHF [6]. While conference presentations may provide access to timely and high-impact insights before their subsequent publication in the literature, there is no consolidated synthesis of this rapidly evolving evidence base. Given the acute and outbreak-prone nature of VHF, recent Ebola outbreaks have highlighted the need to accelerate the development, evaluation, and deployment of medical countermeasures, including therapeutics and vaccines [5]. Timely identification and characterization of emerging evidence on VHF interventions is particularly important, supporting the need for a structured synthesis of these data.

Given the ongoing burden of viral hemorrhagic fevers (VHFs) globally, we specifically sought to update intelligence on targeted pharmacological interventions for the prevention and treatment of VHFs presented at the 74th Annual Meeting of the American Society of Tropical Medicine and Hygiene (ASTMH), held in Toronto, Ontario, Canada in November 2025. Given the acute, outbreak-prone nature of VHF and the need for rapid translation of emerging data into clinical and public health practice, a structured synthesis of these data is warranted.

## 2. Methods

### 2.1. Protocol and Data Source

A systematic review of all poster and oral presentations in the ASTMH Program and List of Symposia was conducted in accordance with the Preferred Reporting Items for Systematic Reviews and Meta-Analysis (PRISMA) guidelines [7,8] (Table S1). The systematic review is registered with the PROSPERO International Prospective Register of Systematic Reviews, protocol CRD420261517212.

### 2.2. Eligibility Criteria

We included all studies that reported on either the use of vaccines and chemoprophylactic agents for prevention of VHFs in people of any age, sex, or geographic place of residence, or the use of targeted biologic therapies for treatment of VHFs in humans of all demographic spectra. All study designs were included, including case reports and other observational designs; as such, some preventive or therapeutic interventions reported would have had no comparator arm. Exclusion criteria included studies that focused exclusively on non-targeted interventions such as infection prevention and control measures or supportive care alone (e.g., intravenous fluids, analgesics, etc.), modeling studies, and non-human animal studies. Additionally, studies that were registered in either the Program or List of Symposia but were not ultimately presented in person at the conference were excluded.

### 2.3. Outcome Measures

Primary outcomes of interest included: (i) measures of immunogenicity for preventative interventions; (ii) incidence of VHF among those exposed; (iii) morbidity and mortality; and (iv) safety, toxicity, and tolerability of preventative and therapeutic interventions amongst VHF-exposed and/or infected individuals. Primary outcomes were synthesized quantitatively and qualitatively where feasible. Secondary outcomes, where available, included: (i) hospitalization length-of-stay and resource utilization; (ii) cost and economic outcomes; (iii) health equity and human rights data; and (iv) measures of feasibility, acceptability, and accessibility. Thematic analysis of secondary outcomes was undertaken to elucidate major challenges in the VHF ecosystem that were reported along with emerging evidence.

### 2.4. Search Strategy

The search strategy was implemented using the ASTMH online conference interface that listed all poster and oral presentations using the following combinations of search terms: (viral hemorrhagic fever OR VHF OR filovirus OR arenavirus OR bunyavirus OR nairovirus OR phenuvirus OR paramyxovirus OR Ebola OR EVD OR Sudan virus OR Bundibugyo OR Tai Forest OR Ravn OR Marburg OR Lassa OR Lujo OR Argentinian hemorrhagic fever OR Junín OR Bolivian hemorrhagic fever OR Machupo OR Chapare OR Sabia OR Guanarito OR Crimean-Congo hemorrhagic fever OR CCHF OR Hantavirus OR Hanta OR Hemorrhagic Fever with Renal Syndrome OR HFRS OR Rift Valley fever OR Severe fever with thrombocytopenia syndrome OR SFTS OR Nipah OR Hendra OR Henipavirus OR Yellow Fever OR Dengue hemorrhagic fever OR Alkhurma hemorrhagic fever OR Kyasanur Forest disease OR Omsk hemorrhagic fever) AND (vaccination OR vaccine OR chemoprophylaxis OR prevention OR preventative OR monoclonal antibody OR mAb OR antibody cocktail OR Zmapp OR Ebanga OR INMAZEB OR Regeneron OR biologic OR antiviral OR remdesivir OR favipiravir OR convalescent plasma OR ribavirin OR treatment OR therapeutics OR experimental therapeutic).

### 2.5. Study Selection, Data Extraction, and Synthesis

Titles and abstracts were screened for inclusion before the start of the conference, and eligible symposia, oral presentations, and poster abstracts were attended in person to conduct data extraction. Oral presentations that reviewed advances in prevention or treatment of VHFs but did not present primary data were “hand-searched” for references to primary data on presenter slides. Title and abstract screening, full-text screening, and data extraction were performed by two independent reviewers, with arbitration of discrepancies by a third independent reviewer. Following data extraction, a table was generated with study characteristics and outcomes (Table 1) and data were narratively synthesized. Given the heterogeneity in study designs, interventions, outcome measures, and reporting from the available studies, formal quantitative analysis of outcomes (e.g., meta-analysis) was not conducted.

### 2.6. Risk of Bias Assessment

Comprehensive risk of bias (ROB) assessment forms were designed based on the Joanna Briggs Institute (JBI) critical appraisal tools, with each study design matched to the corresponding JBI checklist [9,10,11]. The forms were completed by two independent reviewers following data extraction, with discrepancies arbitrated by a third, independent reviewer and/or through consensus discussion. Given the heterogeneity in study designs, interventions, and outcome measures, in addition to variable reporting based on the preliminary or final nature of the presented data, the Grading of Recommendations, Assessment, Development, and Evaluation (GRADE) framework [12,13] could not be rigorously applied to rate the certainty of evidence.

## 3. Results

### 3.1. Search Results

**Figure 1:**
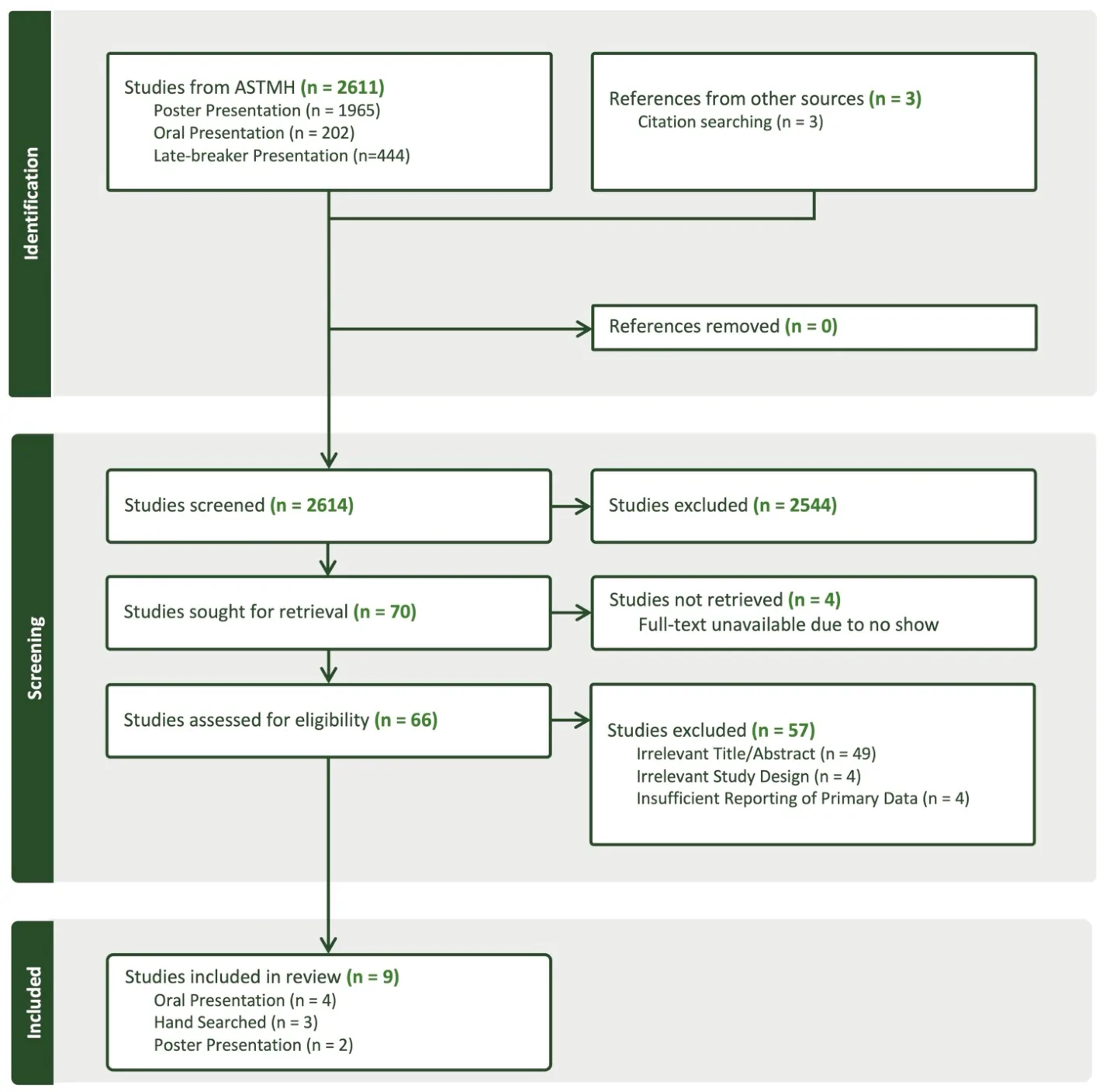
PRISMA Flow Diagram.

The search strategy identified nine studies for ultimate inclusion and synthesis. Of the nine studies, there were two poster presentations, four oral presentations, and three hand-searched references. One of the oral presentations that presented preliminary findings subsequently published full results in a peer-reviewed journal; in this case, the peer-reviewed publication was extracted in place of the oral presentation at ASTMH. The three hand-searched references were obtained from two additional oral presentations [14,15] that did not present novel primary data but reviewed new and emerging data that met our inclusion criteria. A comprehensive summary of the screening process and reasons for presentation or abstract exclusion is available in the PRISMA Flow Diagram shown in Figure 1.

### 3.2. Included Studies

Of the nine studies and presentations ultimately included and synthesized, seven evaluated pharmacological prevention of VHFs and two evaluated experimental and/or licensed pharmacotherapeutics, with two studies contributing evidence to both categories. One observational study and one narrative review provided contextual evidence on vaccine readiness and outbreak preparedness. The included evidence comprised four phase 1 trials, one phase 2 randomized controlled trial, one systematic review, one cross-sectional study, one narrative review, and one case series and retrospective epidemiologic and clinical analysis. Preventative interventions included vaccines targeting Sudan virus, ebolavirus, Nipah virus, and Marburg virus, along with ribavirin and remdesivir for post-exposure chemoprophylaxis. Therapeutic evidence included the monoclonal antibody m102.4 and ribavirin for henipaviruses, along with monoclonal antibody MBP091 and remdesivir for Marburg virus disease. Across studies, reported outcomes focused primarily on safety and tolerability, immunogenicity and durability of antibody responses, morbidity and mortality, and operational considerations related to outbreak preparedness and rapid deployment.

**Table 1:** Study characteristics.

Author (Year)	Study Design	Type of VHF	Population Characteristics	Intervention	Licensure Status of Intervention	Outcomes
***Presented at ASTMH, Unpublished***						
Gilbert et al. (2025) [16]	Phase 1 Trial	Nipah virus	Sentinel cohort including 6 adults and main cohort including 45 adults	ChAdOx1 NipahB vaccine and saline placebo (*n* = 5)	FDA: Not approved; EMA: Not authorized (PRIME designation granted 2025); Health Canada: Not approved.	**Primary:** ChAdOx1 NipahB induced binding and neutralizing antibody responses (40/40 seroconverted after dose 1, PNA detectable in 5/6 after dose 1 and 6/6 after dose 2), with responses boosted by a second dose and maintained through available follow-up (~day 140). No serious adverse events were reported. Asymptomatic thrombopenia occurred in 2 volunteers, including 1 potentially spurious result that resolved on repeat testing. **Secondary:** clinical efficacy assessment was not considered feasible because of sporadic/low Nipah virus case numbers and absence of an established correlate of protection—the proposed strategy is to establish an immunological correlate of protection through non-human-primate challenge studies for use as a surrogate efficacy endpoint.
Heppner et al. (2025) [17]	Phase 1 Trial	Nipah virus	A. 61 healthy adults and B. 120 healthy adults	Nipah Virus Vaccine (PHV02); A: One IM dose of 1.0 × 10^5^: 1.0 × 10^6^: 1.0 × 10^7^: placebo (1:1:1:1 pfu) with 5.0 × 10^8^ booster at 1 yr; B: Two IM doses of 2.5 × 10^6^: 2.5 × 10^7^: 2.5 × 10^8^: placebo (1:1:1:1 pfu) 28 days apart	FDA: Not approved; EMA: Not authorized; Health Canada: Not approved.	**Primary:** all vaccine doses were well tolerated. Neutralizing antibody seroconversion occurred after single-dose vaccination and increased following booster vaccination, reaching 96% in the two highest-dose groups by day 57. A rapid anamnestic response following a booster at 1 year demonstrated immunologic memory.
Malkevich et al. (2025) [18]	Phase 1 Trial	Ebola–Sudan virus (SUDV)	36	rVSVΔG-SEBOV-GP vaccine: IM dose of 2 × 10^6^–2 × 10^8^ pfu (*n* = 10 at each dose); and placebo (*n* = 6)	FDA: Not approved; EMA: Not authorized; Health Canada: Not approved.	**Primary:** The vaccine was safe and well tolerated across all dose levels, with no serious vaccine-related adverse events. Transient mild-to-moderate local pain and tenderness were reported, while systemic reactions occurred in 5–12% of participants in the higher-dose groups and resolved within a few days. All vaccinated participants had detectable SUDV-GP-specific IgG by day 28, which remained detectable in all participants at 6 months. Neutralizing activity persisted through day 168. **Secondary:** vaccine study implementation within 5 days of outbreak confirmation demonstrated feasibility of rapid emergency deployment using pre-approved protocols, in-country vaccine availability, and existing collaborations.
Berrian et al. (2025) [19]	Cross-sectional Study	Ebolavirus (EBOV)	1200 participants, including 696 females and 504 males, with a mean age of 40 (18–80) years	Survey to identify community-based Ebola preparedness and vaccination readiness strategies	N/A	**Secondary:** Outbreak/community preparedness, healthcare infrastructure, affordable healthcare, trust in healthcare systems, and perceived vaccine safety/effectiveness were associated with vaccination likelihood/uptake. Vaccine hesitancy was attributed to inadequate information, mistrust, and cultural beliefs. Qualitative findings emphasized community engagement, education, trusted communication, local leadership, training, and resource preparedness as important for vaccine uptake and future outbreak preparedness.
***Unpublished at Time of ASTMH Presentation, Now Published***						
Tremblay et al. (2026) [20]	Randomized Control Trial, Phase 2	Ebolavirus (EBOV)	251 enrolled, with 250 receiving intervention. Adults and adolescents living with HIV with CD4 counts ≥200 and undetectable viral load. Sex: 120 male and 130 female. Age: median 52.0 (IQR 46.0–56.0).	Ebola Virus Vaccine V920 rVSVΔG-ZEBOV-GP (rVSV-ZEBOV-V) and placebo. Enrolled 4:1 vaccine (*n* = 201) to placebo (*n* = 49). Cohorts 1–4 received one dose of vaccine or placebo. Cohort 5 received two doses of vaccine or placebo, 56 days apart. Combined cohort = cohorts 1–5, single dose of vaccine or placebo.	**ERVEBO**—FDA: Approved (2019); EMA: Authorized (2019; full authorization 2021); Health Canada: Approved (2022).	**Primary Immunogenicity:** In the combined cohort, GP-ELISA GMT was 42-fold higher at 28 days after vaccine (*n* = 198) versus placebo (*n* = 48) (GMTR 42.90, 95% CI 31.49–58.43, *p* < 0.0001). PRNT GMFI was 11.51 (9.82–13.50) in vaccine recipients versus 1.00 (1.00–1.00) in placebo recipients. In cohort 5, GP-ELISA GMT was >200-fold higher at 28 days after the second dose of vaccine (*n* = 40) versus placebo (*n* = 10) (GTMR 208.66, 95% CI 115.57–376.72, *p* < 0.0001). PRNT GMFI was 86.64 (69.85–107.46) in vaccine recipients versus 1.00 (1.00–1.00) in placebo recipients. **Secondary Immunogenicity:** Immune responses were maintained through day 365 (*n* = 197); in participants receiving a second vaccine, GP-ELISA GMTs declined by day 180 and were similar to single-dose GMTs at day 365. **Primary Safety:** Most common adverse events in the vaccine group were injection site pain (130/201), headache (109/201), fatigue (91/201), and joint pain (50/201); all more common in the vaccine group. Fever, rash, and blisters/vesicles up to day 42 after vaccination were uncommon (<5%). Unsolicited adverse events up to day 42 were not different between vaccine and placebo (51% versus 45%, *p* = 0.52). No “clinically significant change” in hematologic or biochemical lab parameters. No vaccine-related SAEs through day 365. **Secondary Safety:** VSV viremia following the first dose of vaccine was detected in 137/200 (67%) on day 3, 2/198 (1%) on day 28, and no viremia on days 14 or 42. VSV viremia following the second dose of vaccine was detected in 3/40 (8%), with 1/40 on day 59 (three days after second vaccine) and 2/40 on day 98 (42 days after second vaccine). Saliva shedding following the first dose of vaccine occurred in 33/200 (17%) on day 7 and 21/200 (11%) on day 14, with no shedding on day 28 or 42. Urine shedding following the first dose of vaccine occurred in 1/200 on day 7. There was no saliva or urine shedding following the second dose. No skin vesicle fluid, joint fluid, or skin biopsy were collected. Six SAEs during the study period, none related to the vaccine. No “clinically significant change” in CD4 count or viral load.
***Previously Published Literature, Reviewed in ASTMH Presentations***						
Chan et al. (2025) [21] ^a^	Systematic Review	Hendra and Nipah Viruses	Eight articles: one RCT, one review article, 5 case reports or series, one observational open-label study	Monoclonal antibody 102.4 (m102.4) as therapeutic; Ribavirin as therapeutic and post-exposure chemoprophylaxis	m102.4: investigational only, not licensed (FDA, EMA, Health Canada). Ribavirin: off-label use of licensed product (FDA, EMA, Health Canada)	**m102.4:** Two studies (therapeutic). One phase 1 RCT for safety/tolerability/immunogenicity. One review article describing compassionate therapeutic use in India, with no outcome details. **Ribavirin:** Six studies (one preventative, five therapeutic). Three case reports or series for treatment of NiV. One case series for treatment of HeV. One observational, non-randomized comparative study for treatment of NiV. One case series for post-exposure chemoprophylaxis of NiV.
Hamer et al. (2023) [22] ^b^	Phase 1 Trial	Marburg virus	40 healthy adults, including 25 females and 15 males, with a mean age of 34.9 (19–48) years	Chimpanzee adenovirus type 3-vectored Marburg virus vaccine (cAd3-Marburg): IM dose at 1 × 10^10^ pu (*n* = 20) and 1 × 10^11^ pu (*n* = 20)	FDA: Not approved; EMA: Not authorized; Health Canada: Not approved.	**Primary:** vaccine was safe and well tolerated with no serious vaccine-related adverse events, including mild injection site pain/tenderness (27/40 [68%]), malaise (18/40 [45%]), headache (17/40 [43%]), and myalgia (14/40 [35%]). Transient asymptomatic decreases in WBC count occurred in 4/40 (10%) and severe fever in 1/40 (3%), which resolved within 1 day. Glycoprotein-specific antibody responses developed in 95% of participants after a single vaccination and remained elevated at 48 weeks. **Secondary:** Findings supported potential emergency deployment and outbreak-response use of the vaccine.
Njenga et al. (2025) [23]	Narrative Review	Ebola—Sudan virus (SUDV)	Two distinct outbreaks: 2022–2023 (142 cases, 55 fatalities) and 2025 (14 cases, 4 fatalities)	N/A	N/A	**Secondary:** Recommendations for vaccine studies in the setting of VHF outbreaks include “pre-approved generic protocol” and “investigational ready reserve vaccines, therapeutics.” In the initial 2022–2023 outbreak, the vaccine study was “time-barred” and not conducted. Protocol approval took 13 weeks, and outbreak was declared over after 16 weeks. In the 2025 outbreak, vaccine study was implemented 4 days after outbreak was confirmed and 131 contacts were vaccinated.
Nsanzimana et al. (2025) [24] ^b^	A. Case Series and B. Retrospective Epidemiologic and Clinical Analysis	Marburg virus	A. 66 laboratory-confirmed MVD cases (21 female and 45 male, with a median age of 31.5 years) with 6340 persons tested overall; B. 1710 frontline workers and high-risk contacts vaccinated as preventive outbreak response; >150 HCWs receiving remdesivir as post-exposure prophylaxis	A. Remdesivir IV (*n* = 52); MBP091 (*n* = 10); B. ChAd3-MARV vaccine (*n* = 1710) and Remdesivir PO (*n* ≥ 150)	FDA: Not approved; EMA: Not authorized; Health Canada: Not approved. Remdesivir is a licensed medication that was used off-label for MVD.	**Primary:** Overall case fatality rate was 23% (15/66). Remdesivir therapeutic: 3/52 died (6%) compared to 12/14 (86%) who did not receive remdesivir. MBP091 therapeutic: 2/10 died (20%), compared to 13/56 (23%) who did not receive MBP091. Formal efficacy assessment was not possible. CFRs by therapeutic do not take into account severity of illness, viral load, comorbidities, supportive care, etc. No immediate drug-related adverse events were identified with remdesivir or MBP091. A total of 1710 frontline workers and high-risk contacts received the ChAd3-MARV vaccine; the effect of the vaccine on transmission efficacy was not assessed directly. No serious vaccine-related adverse events were reported during emergency ChAd3-MARV vaccine rollout. Over 150 HCWs received remdesivir post-exposure prophylaxis; no follow-up data reported. **Secondary:** feasibility: vaccine was deployed within 13 days of outbreak declaration; rapid initiation of a phase 2 clinical trial for vaccine safety and immunogenicity; four patients receiving emergency MBP091 were enrolled in an RCT coordinated by WHO.

^a^ Hand-searched from references during oral presentation “Nipah therapeutics—a holistic approach” by Olliaro [18], ^b^ Hand-searched from references during oral presentation “Marburg Virus: What’s New” by Kortepeter [14]; **AKI:** acute kidney injury, **CI:** confidence interval, **EMA:** European Medicines Agency, **FDA:** U.S. Food and Drug Administration, **GMT:** geometric mean titer, **GP-ELISA:** glycoprotein enzyme-linked immunosorbent assay, **HCW**: healthcare worker, **IgG:** immunoglobulin G, **IM:** intramuscular, **IV:** intravenous, **kg:** kilogram, **mg:** milligram, **MVD:** Marburg virus disease, **PFU:** plaque-forming unit, **PNA:** pseudovirus neutralization assay, **PO:** per os (by mouth), **PRIME:** PRIority MEdicines, **PRNT:** plaque reduction neutralization test, **pu:** particle unit, **RCT:** randomized control trial, **SAE:** serious adverse events, **VSV:** vesicular stomatitis virus, **WBC:** white blood cell, **WHO**: World Health Organization.

### 3.3. Risk of Bias Assessment

**Figure 2:**
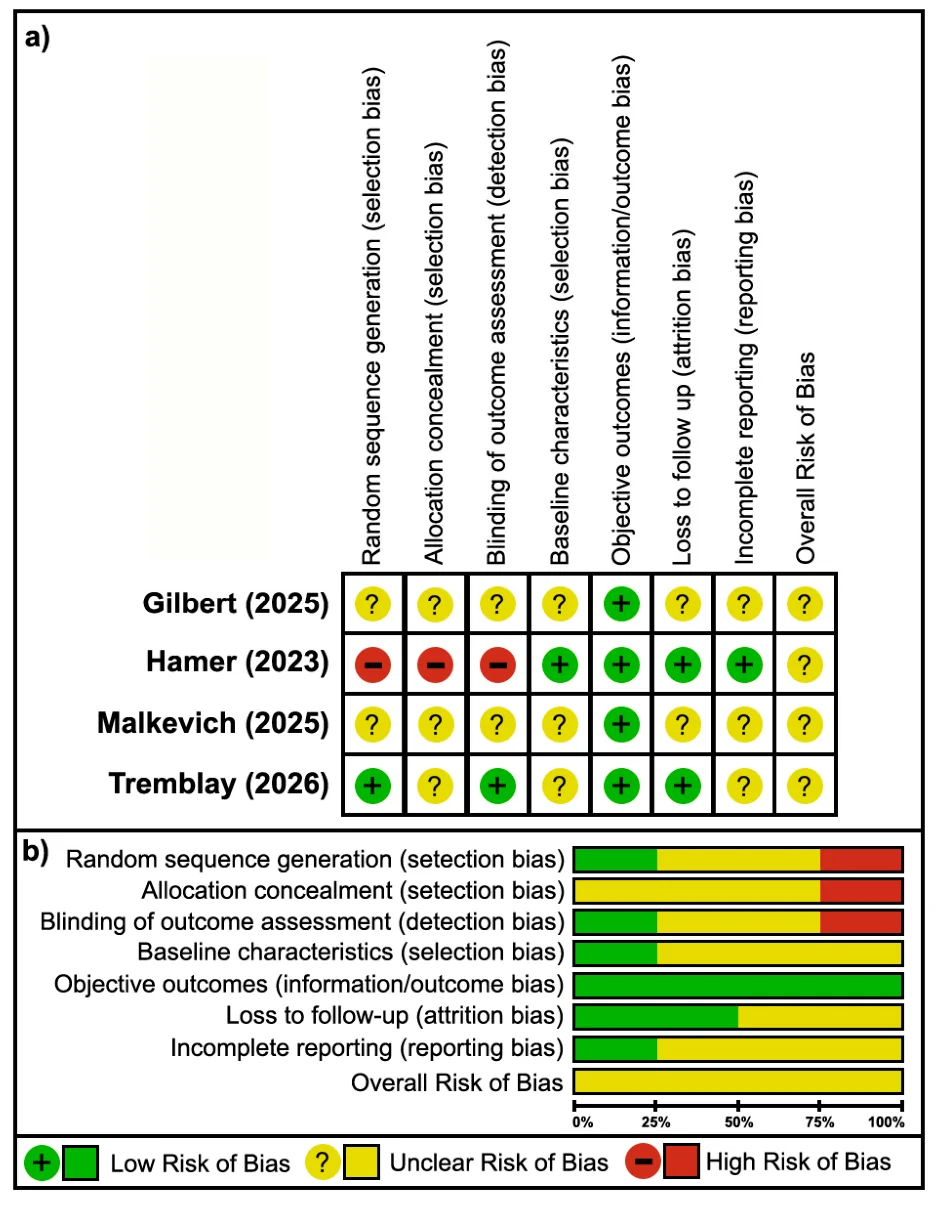
Risk of bias heat map. Individual study risk of bias (**a**), and aggregate representation of risk of bias degrees (**b**). References [16,18,20,22].

Risk of bias heat maps were generated for randomized trials for preventative measures (Figure 2). Green indicates low risk of bias, red indicates high risk of bias, and yellow/amber indicates unclear or indeterminate risk (where conference abstracts or preliminary presentations did not provide sufficient methodological detail for a definitive judgment). The granularity of the ROB assessment was limited by the level of methodological detail reported in the presented studies, as certain studies reported data in the interim or preliminary form, without a full description of the study methodology. Therefore, the predominance of unclear assessments reflects incomplete reporting of randomization, allocation concealment, blinding, baseline comparability, follow-up, and outcome reporting in the preliminary conference data rather than demonstrated methodological deficiencies. This is reflected in the uncertainty of the risk of bias across various domains of several included studies. Given that only two studies detailed therapeutic interventions (one systematic review [21] and one retrospective epidemiologic and clinical analysis and case series [24]), a heat map for therapeutic measures was not generated. The systematic review was assessed to have a low risk of bias.

### 3.4. Narrative Synthesis—Preventive Interventions

Reported preventive interventions were evaluated for Sudan virus, ebolavirus, Nipah virus, and Marburg virus, with the evidence base consisting predominantly of early-phase vaccine studies. The vaccines included in the studies were the ChAdOx1 NipahB vaccine and Nipah Virus Vaccine PHV02 for Nipah virus, cAd3 vaccine for Marburg virus, rVSVΔG-ZEBOV-GP vaccine (ERVEBO) for ebolavirus, and rVSVΔG-SEBOV-GP vaccine for Sudan virus. There were two references to post-exposure chemoprophylaxis: oral ribavirin for Nipah virus and oral remdesivir for Marburg virus.

Five studies reported on markers of immunogenicity following vaccination. In a phase 1 trial of rVSVΔG-SEBOV-GP vaccine against Sudan virus, all participants developed detectable SUDV-GP-specific IgG by day 28, with responses remaining detectable at six months, and neutralizing activity persisting through day 168 [18]. In a phase 2 randomized control trial of rVSVΔG-ZEBOV-GP vaccine (ERVEBO) in adults and adolescents living with HIV with undetectable viral loads and CD4 counts ≥200, vaccine recipients demonstrated substantially greater GP-ELISA and plaque reduction neutralization responses than placebo recipients at day 28 following vaccination [20]. GP-ELISA geometric mean titers (GMTs) were 42-fold higher after vaccine versus placebo (GMT ratio 42.90, 95% CI 31.49–58.43, *p* < 0.0001). In a cohort of adults and adolescents receiving a second vaccine dose 56 days following the initial vaccine dose, the GP-ELISA GMT was over 200-fold higher at 28 days after the second dose compared to placebo (GMT ratio 208.66, 95% CI 115.57–376.72, *p* < 0.0001). Immune responses were maintained through day 365. In participants receiving a second vaccine, GP-ELISA GMTs declined by day 180 and were similar to single-dose GMTs at day 365. In a phase 1 trial of the ChAdOx1 NipahB vaccine against Nipah virus, ChAdOx1 NipahB vaccine induced binding antibody seroconversion in all evaluable participants (40/40) and neutralizing antibody response in 5/6 participants after the first dose, with neutralizing responses detectable in 6/6 participants following a second dose [16]. Antibody responses were boosted following the second dose and maintained through available follow-up data (around day 140). In a phase 1 trial of the PHV02 vaccine against Nipah virus, PHV02 similarly induced neutralizing antibody responses, with seroconversion reaching 96% in the two highest-dose groups by day 57 and an anamnestic response observed following booster vaccination at one year [17]. In a phase 1 trial of the cAd3-Marburg vaccine, induced glycoprotein-specific antibody responses were detected in 95% of participants after a single dose, with responses remaining elevated through 48 weeks [22]. A separate epidemiologic review described the use of 1710 doses of ChAd3-Marburg vaccine in the 2024 Marburg outbreak in Uganda; however, there was no formal efficacy assessment conducted [24].

Two studies briefly mentioned pharmacologic chemoprophylaxis for VHFs, oral ribavirin for Nipah virus, and oral remdesivir for Marburg virus. The retrospective epidemiologic analysis of the 2024 Marburg outbreak in Uganda described over 150 courses of oral remdesivir post-exposure prophylaxis prescribed for healthcare workers; no follow-up data on Marburg incidence was reported [24]. A systematic review of Nipah virus therapeutics mentioned one case series of ribavirin post-exposure chemoprophylaxis of NiV [21]. The systematic review did not include any pooled data or meta-analysis on pharmacologic post-exposure prophylaxis. Overall, the preventive studies primarily reported safety, tolerability, and immunogenicity outcomes of vaccines in phase 1 and phase 2 trials.

There was only one study that mentioned the use of vaccination in a real-world outbreak setting, but an efficacy assessment was not feasible during the emergency-use vaccine rollout.

Across the included studies, vaccines were generally well tolerated, with no serious vaccine-related adverse events reported. Adverse events were primarily transient local or systemic reactions. The rVSVΔG-SEBOV-GP vaccine was associated mainly with mild-to-moderate local injection site reactions and lower rates of systemic symptoms at higher doses [18]. The most common adverse events following the rVSVΔG-ZEBOV-GP vaccine (ERVEBO) were injection site pain (65%), headache (54%), fatigue (45%), and joint pain (25%), all of which were more common in the vaccine group [20]. Following vaccination, transient VSV viremia was detected in 137/200 (67%) on day 3, dropping to 2/198 (1%) on day 28 and no viremia on day 42. VSV viremia following a second dose of vaccination was rare. Viral shedding in saliva occurred in 17% on day 7 and resolved by day 28, and viral shedding in the urine occurred in one individual at day 7. There were no clinically significant changes reported in hematologic parameters, biochemical lab parameters, viral load, or CD4 count, although neither the values nor the formal statistical analysis were reported. In the ChAdOx1 NipahB study, two participants developed asymptomatic thrombocytopenia, including one potentially spurious result that resolved on repeat testing [16]. Following cAd3-Marburg vaccination, the most common reported side effects were injection site pain (68%), malaise (45%), headache (43%), and myalgia (35%) [22]. There was a transient decrease in the white blood cell count in four participants, and one participant developed severe fever that resolved within one day. No serious vaccine-related adverse events were reported during the emergency ChAd3-MARV vaccine rollout in Rwanda [24].

### 3.5. Narrative Synthesis—Therapeutic Interventions

Therapeutic interventions targeting henipaviruses and Marburg virus disease were represented by a systematic review of the current landscape of Nipah therapeutics along with a retrospective case series on the use of emergency therapeutics for Marburg disease during the 2024 Uganda outbreak. The systematic review identified two therapeutics with human use for NiV: monoclonal antibody 102.4 (m102.4) and ribavirin [21]. For m102.4, there was one phase 1 RCT for immunogenicity, safety, and tolerability, along with one review article describing compassionate use in an outbreak in India, with no outcome details reported. For ribavirin, there were five therapeutic studies identified, which included three case reports or series for treatment during NiV outbreaks, one case series for treatment of Hendra virus, and one observational, non-randomized comparative study for treatment of NiV in the initial Malaysian outbreak. The systematic review did not include any pooled data or meta-analysis on pharmacologic post-exposure prophylaxis due to heterogeneity in study designs, outcome measures, and reporting. The individual studies identified by the systematic review were not reviewed at ASTMH and, as such, are not detailed here. During the 2024 Marburg virus disease outbreak in Rwanda, remdesivir and MBP091 were administered as emergency investigational therapeutics to patients with laboratory-confirmed disease [24]. The overall case fatality rate of the outbreak was 23%. Fifty-two patients received intravenous remdesivir, including all critically ill patients requiring supplemental oxygen or end-organ support. Three of these 52 patients died (6%), in comparison to 12 deaths in the 14 patients that did not receive remdesivir (86%). Ten patients received MBP091, six under the expanded-access protocol and four as part of a WHO randomized control clinical trial. Two of these ten patients died (20%), in comparison to 13 deaths in the 56 patients who did not receive MBP091 (23%). Given that the drug was used primarily on an emergency basis without randomization, no formal efficacy assessment was deemed possible by the authors. For both therapeutics, there were no immediate drug-related adverse events identified.

### 3.6. Thematic Analysis of Secondary Outcomes

Thematic analysis of reported secondary outcomes indicated that major emerging themes in the field of VHF relate to operational challenges, including the importance of outbreak preparedness, healthcare infrastructure, regulatory readiness, and rapid deployment capacity for effective implementation of VHF countermeasures. Multiple vaccine studies noted issues with the feasibility of conducting clinical efficacy assessments, for reasons such as sporadic and low case numbers, lack of established correlates of protection, and prioritization of the emergency use of vaccines in real-world outbreak settings rather than controlled clinical efficacy assessments [16,24]. Despite the limitations in conducting real-world efficacy trials, multiple studies did note that rapid vaccine rollout in outbreak settings is feasible. In the 2024 Marburg outbreak in Uganda, the vaccine was deployed within 13 days of outbreak declaration, with the rapid initiation of a phase 2 clinical trial for vaccine safety and immunogenicity [24]. In the 2025 Sudan virus outbreak in Uganda, a vaccine study was implemented four days after outbreak confirmation [18,23]. These scenarios highlight the importance of pre-approved protocols and vaccine reserves in outbreak response. In the 2022–2023 Sudan virus outbreak in Uganda, in comparison, lack of pre-approved protocols and vaccine infrastructure resulted in a 13-week protocol approval window [23]. The outbreak was declared over by 16 weeks, and no vaccines were distributed during that outbreak as a result. Encouraging phase 1 results on immunogenicity, safety, and tolerability support emergency deployment of vaccines for use in outbreak response [22]. Despite the successful emergency use of vaccines in recent outbreaks, it is important to consider local and context-specific factors that may influence vaccine uptake. A cross-sectional study of ebolavirus preparedness and vaccination readiness identified outbreak/community preparedness, healthcare infrastructure, affordable healthcare, trust in health systems, and perceived vaccine safety and effectiveness as factors that determine vaccination uptake [19]. Vaccine hesitancy was attributed to factors such as inadequate information, mistrust in healthcare, and cultural beliefs [19]. Qualitative analysis revealed community engagement, education, trusted communication, local leadership, training, and resource preparedness as key facets for vaccine uptake and outbreak preparedness [19].

## 4. Discussion

We synthesized emerging and evolving data on pharmacointerventions for VHF through a rapid systematic review of evidence presented at thepremiere tropical medicine conference globally, the Annual Meeting of the American Society of Tropical Medicine and Hygiene. The overall quality of presented evidence was uncertain, largely due to the lack of granularity of available methodological detail reported. Two of the four clinical trials were presented at ASTMH as interim or preliminary data and have not been subsequently published [16,18]. The lack of availability of the complete methodology precludes comprehensive assessment of the risk of bias in key domains such as random sequence generation, allocation concealment, baseline characteristics, loss to follow-up, and complete reporting of outcomes. Although these trials were both reported as blinded (one single-blinded and one double-blinded), there was no information on which parties were blinded or how blinding occurred, thereby further limiting our assessment of detection bias. An additional trial on the cAd3-Marburg vaccine [22] was an open-label trial without randomization, allocation concealment, or blinding, thereby leading to an overall uncertain risk of bias. Finally, a recently published trial on the use of ERVEBO in persons living with HIV had a lack of granularity with respect to allocation concealment, statistical differences between baseline characteristics, and incomplete reporting of several primary and secondary outcomes. Overall, there was an unclear risk of bias with respect to vaccine outcomes.

We acknowledge that the risk of bias assessment will likely change as some of the preliminary and interim data become published in full. Not included in the risk of bias assessment were case series or descriptive retrospective analyses [24] or studies that only reported on secondary outcomes regarding feasibility, tolerability, and acceptability [19,23]. Given that only two studies discussed therapeutic options, with one being a case series and one a descriptive systematic review without pooled outcomes or meta-analysis, a risk of bias heat map was not generated for therapeutics [21,24]. The systematic review on Nipah therapeutics was deemed to be low risk of bias [21].

The largely uncertain risk of bias in these studies reflects important real-world limitations on evaluating targeted preventative and therapeutic measures for VHFs. Given the sporadic and unexpected nature of VHF outbreaks, these measures are often procured on an emergency basis for compassionate or investigational use. Formal clinical efficacy trials in these settings have many barriers, including absence of predetermined clinical correlates, lack of prepositioned vaccines or therapeutics, lack of trial infrastructure, insufficient funding, and ethical dilemmas around the use of placebos. Sporadic outbreaks also contribute to the difficulty of conducting adequately powered studies. Phase 1 trials are often conducted in settings non-endemic for VHFs, and translating studies from these settings to regions that are disproportionately burdened by VHFs remains challenging. Collaborative efforts between pharmaceutical companies, governments, public health agencies, medical personnel, and community leaders are required on both international and local levels in order to ensure not only timely access to preventative and therapeutic measures in outbreak settings, but also to evaluate their efficacy, safety, and tolerability in endemic regions. Data are often released as preliminary or interim results, as reflected in our systematic review, further limiting the ability to evaluate the methodological quality of available and evolving data.

### 4.1. Efficacy of Pharmacointerventions for VHF

Strong immunogenicity was demonstrated through sustained antibody responses and high seroconversion rates in vaccine trials, regardless of pathogen or vector platform used [16,17,18,20,22]. This consistency stands out given that these candidates span four different VHF-causing viruses and three separate vaccine platforms, suggesting immunogenicity itself may be a tractable target across the filovirus and henipavirus families even where clinical efficacy data remain unavailable. Two findings are particularly important. First, the licensed Ebola vaccine V920 retained strong immunogenicity when extended to people living with HIV [20], addressing a population historically excluded from VHF vaccine research and outbreak deployment. Second, in the continued absence of any licensed SUDV countermeasure, even early-phase immunogenicity data [18] carry outsized practical relevance, representing the only available evidence base for a pathogen with no existing licensed alternative. The available evidence on VHF therapeutics is much more limited, comprising only two studies. A recent systematic review on the prioritization of Nipah virus drug candidates for clinical trials identified seven studies with human data on Nipah virus therapeutics, two on monoclonal antibody m102.4, and five on ribavirin [21]. Due to heterogeneity in study designs, outcome measures, and reporting, the authors were unable to pool data or conduct meta-analyses, precluding any quantitative data synthesis on Nipah therapeutics in humans.

A case series of the Uganda Marburg outbreak in 2024 described real-world use of remdesivir and monoclonal antibody MBP091. The case fatality in patients administered remdesivir was notably lower (3/52, 6%) than in those not receiving remdesivir (12/14, 86%); however, formal efficacy assessment was not possible, and the authors acknowledge that other factors may have contributed to the differences in mortality rates. MBP091 use was associated with death in 2/10 patients (20%) compared to 13/56 (23%) that did not receive MBP091; similarly, formal efficacy assessment was not possible [24].

Favipiravir is a broad-spectrum antiviral that has been evaluated across VHF contexts but was not represented among the eligible ASTMH presentations included in this systematic review. Recently, an open-label randomized phase 2 trial in Nigeria compared favipiravir with ribavirin in 41 adults with mild-to-moderate PCR-confirmed Lassa fever; favipiravir achieved the prespecified pharmacokinetic and safety endpoints, with no severe or serious adverse events, although the trial was insufficiently powered to establish a mortality benefit [25]. The use of oral broad-spectrum antivirals during VHF outbreaks remains attractive because of potential ease of administration and stockpiling, but practical use requires pathogen-specific dosing and pharmacokinetic data, reliable diagnostics, supply chains capable of early treatment, and prospective efficacy evaluation rather than extrapolation from in vitro activity.

Combination therapy may further help address limitations of treatment by pairing interventions with complementary mechanisms, for example, a direct-acting antiviral with a neutralizing monoclonal antibody. This concept has moved beyond theory in 2026: WHO prioritized evaluation of remdesivir with monoclonal antibodies for Bundibugyo virus disease, and the PARTNERS adaptive randomized trial is evaluating MBP134, remdesivir, and their combined use [26,27]. Platform and adaptive trial designs are particularly suited to assess sporadic VHF outbreaks because they can preserve a common infrastructure across outbreaks while allowing candidates or combination therapies to be added as evidence evolves. Post-exposure and therapeutic vaccination represent another potential strategy that is under further investigation. Rapidly acting vaccine platforms may have a role after exposure or during very early infection, particularly for filoviruses. Preclinical studies of recombinant vesicular stomatitis virus (rVSV)-based vaccines have demonstrated post-exposure protection against Sudan and Bundibugyo viruses in non-human primate models, suggesting that these platforms may provide benefit before a conventional adaptive immune response is fully established [28,29]. Early protection may involve rapid activation of innate antiviral immunity in addition to subsequent antigen-specific responses. However, post-exposure efficacy has varied according to virus, vaccine construct, timing of administration, and experimental models. Clinical efficacy as treatment of established symptomatic VHF has not been demonstrated. Therapeutic vaccination may be most appropriately developed not as a replacement for antiviral or antibody therapy, but as one component of a combined post-exposure and early-treatment strategy. Importantly, nearly all of the efficacy evidence summarized here consists of immunogenicity or pharmacokinetic surrogates, rather than established clinical protection or treatment efficacy, a limitation explicitly acknowledged by the Nipah ChAdOx1 investigators, given the lack of any established correlate of protection for this pathogen [16]. Until later-phase or outbreak-embedded efficacy data become available, these findings are best read as proof-of-concept rather than evidence of protection or benefit under real-world conditions.

For clinical and public health practice, this evidence supports continued advancement of these candidates toward emergency-use pathways during active outbreaks, particularly for pathogens such as SUDV with no licensed alternative, but does not yet support their substitution for established outbreak-control measures. Future studies should prioritize establishing a correlate of protection for vaccine studies, conducting outbreak-embedded efficacy trials where ethically and logistically feasible, and extending safety and immunogenicity data to pregnant, pediatric, and other populations excluded from the trials reviewed here.

In addition, host-directed antivirals are another potential target against filoviruses. Niemann–Pick C1 (NPC1), an endosomal and lysosomal membrane protein, is an essential intracellular receptor for ebolavirus. After proteolytic priming, cleaved viral glycoprotein binds NPC1 domain C to enable membrane fusion [30,31]. Small molecules that disrupt GP-NPC1 engagement or NPC1-dependent endolysosomal cholesterol trafficking can inhibit ebolavirus infection in vitro. Drug repurposing studies have also shown that itraconazole, which can inhibit NPC1 function, and fluoxetine, which indirectly alters endolysosomal lipid homeostasis, reduce ebolavirus infection in cell culture [32,33]. However, this approach remains preclinical. Since NPC1 performs essential host functions, clinical translation of targeting this transporter will require defining an appropriate therapeutic window to achieve sufficient antiviral activity while concurrently preserving normal cholesterol trafficking to avoid host toxicity.

Since the 2025 ASTMH meeting, the global vaccine pipeline has also further evolved. In 2026, there was an acceleration of Bundibugyo virus vaccine development during the ongoing outbreak, with a ChAdOx1 BDBV candidate entering a first-in-human phase 1 study in the United Kingdom. Moderna’s mRNA-1469 candidate also entered a phase 1 trial in Canada [34,35]. Additional Bundibugyo candidates using rVSV and other platforms are being advanced toward clinical evaluation. Further, Lassa fever vaccine development includes an ongoing phase 2 program for IAVI’s rVSV-based candidate and a phase 1 evaluation of a ChAdOx1 candidate, while Nipah vaccine development has advanced into phase 2 clinical evaluation in affected regions [36].

### 4.2. Safety and Tolerability of Pharmacotherapeutics for VHF

Across Ebola, Nipah, and Marburg virus studies, preventive and therapeutic interventions were generally safe and well tolerated, with adverse events limited almost exclusively to mild, transient, self-resolving reactogenicity and no serious vaccine-related events reported in any controlled trial [16,17,18,20,22]. This consistency across four pathogens and multiple vector platforms is encouraging, though it needs to be weighed against how small and early-phase most of this evidence still is.

Two aspects of the safety evidence merit specific attention. First, safety evidence is beginning to extend to populations historically underrepresented in VHF trials: rVSVΔG-EBOV-GP showed no unexpected safety signal in people living with HIV, including resolution of vaccine-strain viremia by day 42 [20], and the ChAdOx1 NipahB trial enrolled a substantially larger adult cohort than earlier henipavirus candidates [16]. Second, safety monitoring during active outbreak deployment necessarily differs from controlled trial conditions: although no drug- or vaccine-related adverse events were identified during the emergency use of remdesivir, MBP091, and the ChAd3-MARV vaccine in Rwanda’s 2024 Marburg outbreak, this reflects passive surveillance carried out under crisis conditions rather than the systematic monitoring of a phase 1 trial, and should not be read as equivalent reassurance [24].

Taken together, these findings support provisional use of these candidates under emergency authorization during active outbreaks, where the risk–benefit balance favors earlier deployment despite being underpinned by limited safety data; any policy decision extending beyond emergency use should await larger datasets with longer follow-up. Future research should build active, structured adverse event surveillance into outbreak deployment rather than relying on passive reporting, and should evaluate safety specifically in pregnant, pediatric, and immunocompromised populations going beyond the single cohort of persons living with HIV captured in this evidence base.

### 4.3. Operational Challenges in the Care of Patients with VHF

Our findings also highlighted major operational challenges in the approach to VHF, including the importance of outbreak preparedness, healthcare infrastructure, regulatory readiness, and rapid deployment capacity for effective implementation of VHF countermeasures. A determinative factor is the capacity of health systems to deploy countermeasures quickly during outbreaks. In the 2025 SUDV outbreak in Uganda, rapid implementation of a ring vaccination trial was enabled by pre-existing protocols, domestic vaccine availability, and a formed relationship network [23]. In a cross-sectional study of the Ebola response in Liberia, community trust and engagement were factors identified to influence vaccine uptake [19]. Ultimately, establishing logistical, political, social, and public health infrastructure prior to an outbreak will lead to a more effective response.

Related challenges come at the transition from early-phase data to licensure and sustainable access. For pathogens with intermittent and geographically concentrated outbreaks, commercial incentives may be lacking, and it may not be feasible to conduct phase 3 efficacy trials outside of an active outbreak. Progress in this regard will require substantial public and philanthropic investments in manufacturing and stockpiles, standardized protocols and regulatory pathways that can be activated quickly, as well as commitments to generate efficacy data during outbreaks. The 2026 Bundibugyo response provides us a precedent for this model. The WHO convened product-prioritization groups and recommended that investigational vaccines and therapeutics be evaluated within research protocols, while international partners financed accelerated development and trial deployment [26]. These structures can help prevent outbreak countermeasures from remaining indefinitely in early development. Although sustainable pathways to licensure will also require continued post-outbreak data stewardship, the capability for long-term manufacturing, and procurement commitments for products with limited conventional markets.

### 4.4. Clinical Challenges in the Care of Patients with VHF

Notable clinical challenges in the VHF response highlighted include: persistent long-term sequelae among SUDV survivors; substantial mortality across different VHFs; and organ-support requirements in MVD. Although long-term clinical sequelae have been well described across a number of VHFs, this has not been in the context of preventative or therapeutic measures. Among survivors of Uganda’s 2022–2023 SUDV outbreak, more than half went on to develop persistent multi-system sequelae, most pronounced in ophthalmologic, central nervous system, and reproductive domains, and nearly half still had detectable viral RNA in semen or breast milk more than six months after infection [23]. Neither finding could be explained by host immune response. Experimental preventative and therapeutic measures were not ultimately employed during this outbreak, which reflects the larger body of available VHF survivorship literature, where long-term clinical sequelae have not been formally evaluated in the context of preventive or therapeutic interventions. As such, the current evidence base does not inform whether preventative or therapeutic measures modulate long-term sequelae of clinical infection. Survivorship, in effect, remains an outcome domain this evidence base has yet to measure.

Mortality in VHF outbreaks remains substantial despite improving countermeasures. During Rwanda’s 2024 MVD outbreak, the case fatality rate was 23% even with emergency access to investigational therapeutics [24]. Although case fatality rates were much lower in those receiving remdesivir (6%), a formal efficacy assessment was not feasible during the compassionate use of therapeutics, and a multitude of other factors may have influenced mortality rates apart from treatment, including severity of illness, viral load, access to care, quality of available supportive care, and patient comorbidities.

These findings carry direct implications for how outbreak response is planned. Survivorship clinics and long-term follow-up protocols deserve a place alongside acute countermeasure deployment, and critical-care capacity, ventilators, and renal replacement therapy should be treated as core measures of outbreak preparedness, not an afterthought to pharmaceutical stockpiling. Future research should work towards standardized long-term outcome measures for VHF survivors across pathogens, test whether earlier intervention during acute infection reduces chronic morbidity, and determine whether markers such as VP40-specific antibodies have a practical role in monitoring viral persistence.

## 5. Conclusions

This systematic review has provided a rapid, structured synthesis of targeted pharmacological interventions presented as emerging evidence at an expert scientific venue. The nine included studies presented at the 74th Annual Meeting of the American Society of Tropical Medicine and Hygiene demonstrated the ongoing development of preventive and therapeutic countermeasures across various VHFs such as ebolavirus, Nipah virus, and Marburg virus, with findings suggesting promising immunogenicity and generally favorable safety and tolerability across identified interventions. The overall quality of evidence was uncertain, reflecting key challenges in designing and implementing trials to assess safety, immunogenicity, and efficacy of pharmacologic measures for VHFs. By consolidating these findings, this systematic review addresses the current lack of a centralized, accessible summary of emerging VHF intervention evidence. By rapidly organizing and evaluating these data, this work facilitates timely interpretation and application in clinical and public health settings where prompt decision-making is critical to effective interventions as clinical and public health needs evolve rapidly. Continued synthesis of emerging evidence, alongside evaluation of subsequent findings, will be particularly important as interventions continue to be developed and implemented.

## Data Availability

Not applicable as no new data were created or analyzed in this review article.

## Supplementary Materials

The following supporting information can be downloaded at: https://www.mdpi.com/article/10.3390/tropicalmed11100296/s1. Table S1: PRISMA 2020 Checklist.
